# Cost-effectiveness of internet-based HIV screening among gay, bisexual and other men who have sex with men (GBMSM) in Metro Vancouver, Canada

**DOI:** 10.1371/journal.pone.0294628

**Published:** 2023-11-27

**Authors:** Jose A. De Anda, Michael A. Irvine, Wei Zhang, Travis Salway, Devon Haag, Mark Gilbert

**Affiliations:** 1 School of Population and Public Health, University of British Columbia, Vancouver, British Columbia, Canada; 2 Institute of Applied Mathematics, University of British Columbia, Vancouver, British Columbia, Canada; 3 BC Centre for Disease Control, Vancouver, British Columbia, Canada; 4 Faculty of Pharmaceutical Sciences, University of British Columbia, Vancouver, British Columbia, Canada; 5 Centre for Advancing Health Outcomes, Vancouver, British Columbia, Canada; 6 Faculty of Health Sciences, Simon Fraser University, Burnaby, British Columbia, Canada; 7 Centre for Gender and Sexual Health Equity, Vancouver, British Columbia, Canada; Beth Israel Deaconess Medical Center/Harvard Medical School, UNITED STATES

## Abstract

**Background:**

GetCheckedOnline is an internet-based screening service aiming to increase HIV testing among gay, bisexual and other men who have sex with men (GBMSM). We assessed the cost-effectiveness of GetCheckedOnline in its first implementation phase at different uptake scenarios compared to clinic-based screening services alone in Metro Vancouver, Canada.

**Methods:**

From a healthcare payer’s perspective, our cost-utility analysis used an established dynamic GBMSM HIV compartmental model estimating the probability of acquiring HIV, progressing through diagnosis, disease stages and treatment over a 30-year time horizon. The base case scenario assumed 4.7% uptake of GetCheckedOnline in 2016 (remainder using clinic-based services), with 74% of high-risk and 44% of low-risk infrequent testers becoming regular testers in five years. Scenario analyses tested increased GetCheckedOnline uptake to 10% and 15%.

**Results:**

The cost per test for GetCheckedOnline was $29.40 compared to clinic-based services $56.92. Compared with clinic-based screening services, the projected increase in testing frequency with 4.7% uptake of GetCheckedOnline increased the costs by $329,600 (95% Credible Interval: -$498,200, $571,000) and gained 4.53 (95%CrI: 0, 9.20) quality-adjusted life years (QALYs) in a 30-year time horizon. The probability of GetCheckedOnline being cost-effective was 34% at the threshold of $50,000 per QALY, and increased to 73% at the threshold of $100,000 per QALY. The results were consistent in the other uptake scenarios. The probability of GetCheckedOnline being cost-effective became 80% at the threshold of $50,000 per QALY if assuming 5-year time horizon.

**Conclusions:**

GetCheckedOnline is almost half the cost of clinic-based services on a per-test basis. However, increased access to testing should be balanced with risk profiles of patients to ensure the implementation can be a cost-effective strategy for increasing HIV screening among GBMSM in Metro Vancouver. Additional analyses are needed to understand the impact of internet-based screening including screening for other STIs and in other populations.

## Introduction

The prevalence of HIV in British Columbia (BC) in 2016 was estimated to be 239 (range 210 to 268) per 100,000 people [1]. Although new HIV diagnoses in BC have steadily decreased over the past decade, this trend has largely been driven by decreases among people who inject drugs, likely as a result of scaling up effective harm reduction and treatment programs in this population [1, 2]. By contrast, new diagnoses for gay, bisexual and other men who have sex with men (GBMSM) have declined more slowly and are becoming proportionally greater, accounting for 70% of diagnosed HIV cases in 2017 vs 52% in 2008 [1], as diagnoses in other groups are steady or declining.

Optimizing early diagnosis and antiretroviral treatment (ART) is a major public health strategy to reduce HIV morbidity, mortality and transmission [3–6]. In BC, as in many other jurisdictions, individuals are commonly tested for HIV by seeing a healthcare provider (i.e., clinic-based screening services, the standard of care) such as sexually transmitted infection (STI) clinics, where the process typically involves two interactions with a healthcare provider (i.e. pre- and post-test visits) [7]. However, clinic-based testing services may pose barriers to testing for GBMSM subgroups such as people who feel discomfort disclosing their sexual history face-to-face with a provider, people who fear judgment from healthcare providers, or people who are unable to access in-person testing services [8].

New approaches–such as internet-based screening–are needed to reduce these barriers. GetCheckedOnline is an internet-based screening service developed by the BC Center for Disease Control (BCCDC) aiming to increase uptake and frequency of screening, reach populations facing testing barriers, and increase the capacity and efficiency of current services [9]. Clients using GetCheckedOnline create an account, complete a behavioral and clinical questionnaire, and receive test recommendations, via a secure website (https://getcheckedonline.com). The client must print or download a lab form to take to a specimen collection site. Samples are then sent to the BC Provincial Public Health Laboratory, where tests are conducted and results are recorded. If results are positive or indeterminate, clients are contacted by phone or e-mail; negative results are given online. Unlike clinic-based services, GetCheckedOnline clients do not receive personalized pre-test counselling, although educational messages are provided via the website. GetCheckedOnline is integrated with the BCCDC provincial STI clinic, allowing records and follow-up to be harmonized between the two modalities. GetCheckedOnline is publicly funded and free to its clients. The initial or first phase of implementation of GetCheckedOnline was focused on GBMSM in the Vancouver area (from September 2014-January 2016) [9].

Internet-based approaches for STI screening are increasingly deployed as part of strategies to increase testing. Cost-effectiveness studies of internet-based STI screening are scarce, and none have been conducted for HIV, to our knowledge [10, 11]. An economic evaluation of GetCheckedOnline is particularly relevant to inform ongoing implementation of GetCheckedOnline and potential introduction of other internet-based screening programs. The objective of this study is to assess the cost-effectiveness of GetCheckedOnline based on different uptake scenarios, as compared to clinic-based services alone, for HIV screening among GBMSM in Metro Vancouver, Canada, from a healthcare payer perspective. The scenarios are based on the early phase of implementation of the program, including observed initial rates of testing according to demographics and sexual behaviours. The main study aims were to evaluate the program’s cost-effectiveness under the first phase of implementation in Vancouver, where the service is offered alongside existing clinic services.

## Methods

### Model structure

A cost-utility analysis was conducted to assess the cost-effectiveness of GetCheckedOnline, in addition to standard of care, with different GetCheckedOnline uptake scenarios, compared with standard of care only. This analysis used a previously constructed epidemiological model that estimated changes in diagnosis rates among GBMSM in Metro Vancouver based on the implementation or expansion of additional testing services [12], modeling the probability of an HIV-negative individual to become infected with HIV, and then to be diagnosed. The GBMSM population was stratified by sexual behaviours (based on risk of infection: low or high) and testing pattern (based on testing frequency: none or infrequent testing; regular testing: at least once a year; or frequent testing: every 3 months) with rates commensurate with the Metro Vancouver context. In order to model long-term benefits and costs related to HIV screening, we expanded that model, so that individuals progressed through stages of HIV infection. To inform model expansion, we conducted a literature review and consulted GetCheckedOnline program and BCCDC STI clinic staff, to ensure that the model reflects local pathways through HIV-related medical services. Our final model structure was similar to a previously reported analysis assessing ART expansion in British Columbia [13].

**Fig 1:**
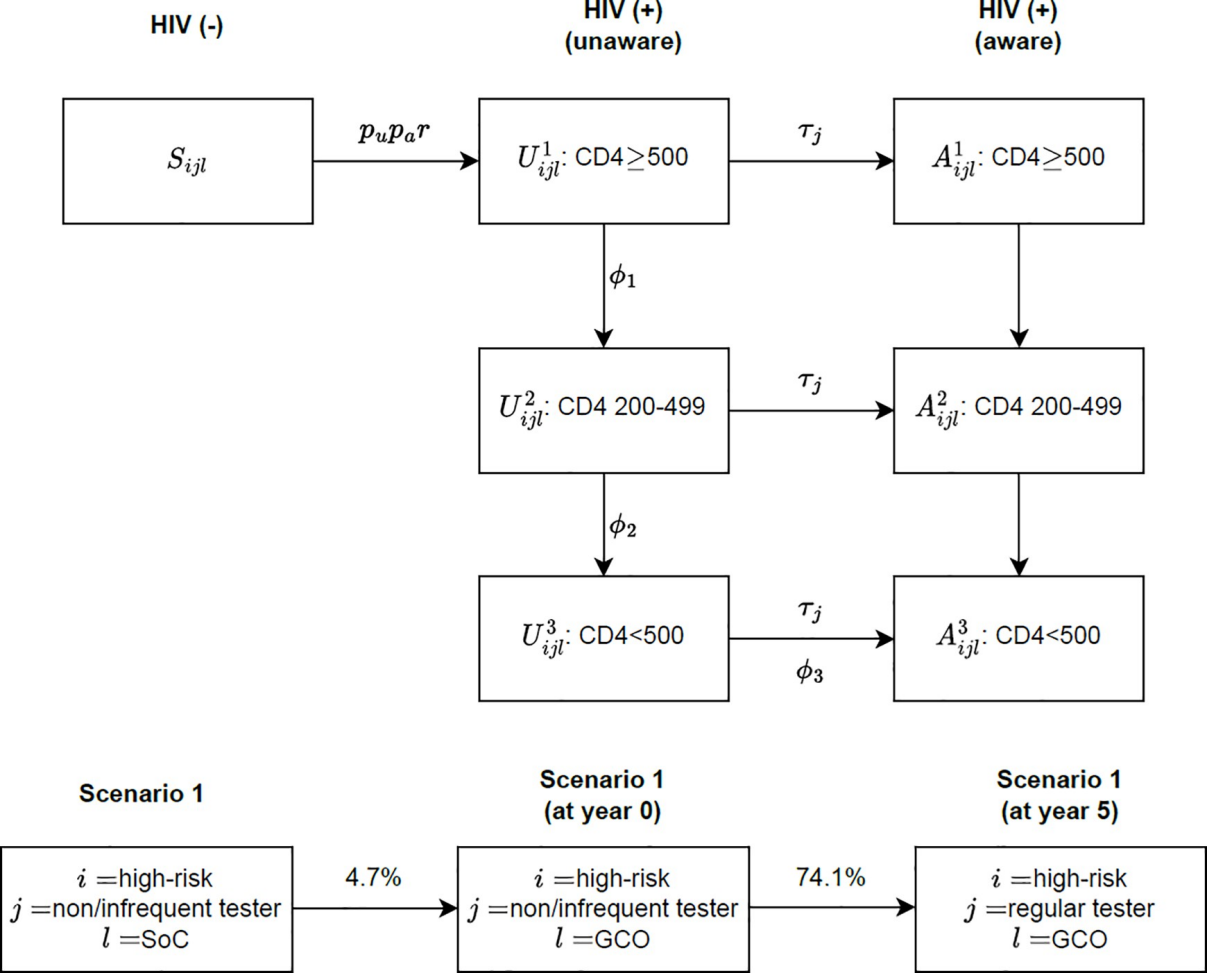
Compartmental model structure of disease progression and main scenario. The top diagram shows the progression of the disease state for each sexual behaviour, testing pattern, and cohort category with the transition from susceptible to unaware dependent also on the proportion of unaware and aware in the population. The bottom diagram shows the initial set-up for scenario one, where there is an immediate transition of individuals in standard of care to the GetCheckedOnline group. This group then transitions from non/infrequent testing to the regular testing group. **Abbreviations:** A—state with HIV and aware; U—state with HIV and unaware; *i*—sexual behaviour; *j*- testing pattern; *l* -cohort; *p*_*a*_—average per-encounter risk among aware individuals; *p*_*u*_—average per-encounter risk among unaware individuals; *r*—average rate of sexual encounter; *τ*_*j*_- rate of testing; *φ* - rate of disease progression; SoC—standard of care; GCO—GetCheckedOnline.

A schematic representation of the model is shown in Fig 1. Each box in the top row represents a health state compartment, and each arrow represents a potential transition. Initially, the population was distributed across compartments according to current HIV prevalence, CD4 counts, and annual diagnosis estimates [14]. Three HIV stages were determined based on CD4 counts. Individuals could progress to a later stage of the disease only while they were unaware of the infection and once diagnosed, they were assumed to stay in the same disease stage at time of diagnosis. Rates of transitioning between health states were given by differential equations (see S1 File which describes the full model and methodology details) incorporating the different parameters influencing such progression (see Table 1). The full model was comprised of two sexual risk behaviour groups and three testing patterns. Testing patterns were applied only to the susceptible and unaware populations. Thus, the model comprised 30 compartmental health states (4×2×3+3×2): 1 susceptible and 3 unaware states (with 2 risk levels and 3 screening behaviours); and 3 diagnosed states (with 2 risk levels).

**Table 1:** Base case model parameters.

Parameter				Unit	Value	95% CI	Source
**Demographics**							
	Total size of sexually active GBMSM			*individuals*	20 000	(12 943, 53 471)	Ref [37]
	Turnover of sexually active population (inverse of duration of being sexually active)			*year* ^*-1*^	0.05		Calibration
**GBMSM initial distribution by sexual behaviour and testing pattern**							
	*Low risk*						
			None or infrequent testing	*%*	16.5	(14.3, 18.7)	Ref [12]
			Regular testing	*%*	43.8	(40.9, 46.7)	Ref [12]
			Frequent testing	*%*	31.3	(28.6, 34.0)	Ref [12]
	*High risk*						
			None or infrequent testing	*%*	1.5	(0.8, 2.2)	Ref [12]
			Regular testing	*%*	4.0	(2.9, 5.1)	Ref [12]
			Frequent testing	*%*	2.8	(1.8, 3.8)	Ref [12]
**Sexual activity**							
	Average time of sexual risk activity			*years*	20	(10, 30)	Ref [38]
	*Rate of sexual encounters*						
			Low risk	year^-1^	51.7	(30.5, 83.5) 4.31 *month*^*-1*^ (2.54, 6.96)	Ref [18, 21, 39]
			High risk	year^-1^	205.5	(122.7, 337.7) 17.1 *month*^*-1*^ (10.2, 28.1)	Ref [18, 21, 39]
	*Transition rate*						
			Low to high risk	year^-1^	0.137	(0.12, 0.16)	Ref [19]
			High to low risk	year^-1^	2.373	(2.28, 2.46)	Ref [19]
**Testing pattern**							
	*Rate of HIV testing*						
			None or infrequent testing	year^-1^	0.00	n/a	Ref [38]
			Regular testing	year^-1^	1.66	n/a	Ref [38]
			Frequent testing	year^-1^	4.00	n/a	Ref [38]
*Transition rate (testing pattern) for SoC to retain testing pattern static*							
			None to regular testing	year^-1^	0.08	(0.06, 0.10)	Ref [38]
			Regular to none testing	year^-1^	0.08	(0.06, 0.10)	Assumption
			Regular to frequent testing	year^-1^	0.29	(0.25, 0.31)	Ref [38]
			Frequent to regular testing	year^-1^	0.29	(0.25, 0.31)	Assumption
**Risk of infection (per encounter)**							
With HIV and unaware							
			Low risk GBMSM	*%*	0.306	(0.219, 0.445)	Ref [18, 21, 39]
			High risk GBMSM	*%*	1.39	(1.01, 1.91)	Ref [18, 21, 39]
With HIV and aware							
			Low risk GBMSM	*%*	0.108	(0.077, 0.155)	Ref [18, 21, 39]
			High risk GBMSM	*%*	0.286	(0.209, 0.394)	Ref [18, 21, 39]
	Treatment effectiveness						
			Infectivity on treatment	*probability*	0		Ref [20]
**Rate of progression**							
	CD4≥500 to CD4 200–499			year^-1^	4.33		Ref [16, 17]
	CD4 200–499 to CD4<200			year^-1^	0.15		Ref [16, 17]
	CD4<200 to hospitalization			year^-1^	0.5		Ref [16, 17]
**Treatment (proportion of GBMSM diagnosed)**							
		Proportion on antiretrovirals		*fraction*	0.840	(0.74, 0.94)	Ref [14]
		Proportion virally supressed		*fraction*	0.710	(0.61, 0.81)	Ref [14]
**Utilities**							
		Susceptible		*utility*	1.00		
		*Infected (Unaware)*					
		CD4≥500		*utility*	0.89	0.85–0.95	Assumption^Ѱ^
		CD4: 200–499		*utility*	0.72	0.70–0.80	Assumption^ϯ^
		CD4<200		*utility*	0.72	0.60–0.75	Ref [24]
		*Diagnosed (off ART)*					
		CD4≥500		*utility*	0.89	0.85–0.95	Ref [24]
		CD4: 200–499		*utility*	0.72	0.70–0.80	Ref [24]
		CD4<200		*utility*	0.72	0.60–0.75	Ref [24]
		*On ART*					
		CD4≥500		*utility*	0.89	0.85–0.95	Assumption^Ѱ^
		CD4: 200–499		*utility*	0.83	0.82–0.87	Ref [24]
		CD4<200		*utility*	0.82	0.82–0.87	Ref [24]
**Screening Costs (2017 CAD)**							
		GCO Screening Test		*per test*	$29.40		BCCDC Estimate
		SoC Clinic-based Test		*per test*	$56.92		BCCDC Estimate
		Positive case management		*per case*	$267.61		BCCDC Estimate
**Annual health States Costs (2017 CAD)**							
		Susceptible		*annual*	$5,409.82		Ref [24]
		*With HIV (Unaware)*					
		CD4≥500		*annual*	$6,698.66		Ref [23, 24]
		CD4: 200–499		*annual*	$10,198.62		Ref [23, 24]
		CD4<200		*annual*	$14,294.73		Ref [23, 24]
		*Diagnosed (off ART)*					
		CD4≥500		*annual*	$7,790.17		Ref [23]
		CD4: 200–499		*annual*	$13,118.04		Ref [23]
		CD4<200		*annual*	$20,260.69		Ref [23]
		*On ART*					
		CD4≥500		*annual*	$26,180.25		Ref [13, 23]
		CD4: 200–499		*annual*	$29,544.32		Ref [13, 23]
		CD4<200		*annual*	$38,267.15		Ref [13, 23]

^Ѱ^ Patients with HIV who remain on CD4≥500 will maintain the same utility regardless of its treatment or disease awareness status.

^ϯ^ Unaware patients with CD4 200–499 will have the same utilities as those diagnosed but not under treatment.

**Abbreviations:** ART–antiretroviral; BC–British Columbia; BCCDC–BC Center for Disease Control; CAD–Canadian dollars; CD4 –Cluster of differentiation 4; CI–Credible interval; GBMSM–Gay, bisexual, and other men who have sex with men; GCO–GetCheckedOnline; SoC–Standard of Care.

Model validation and calibration methods were explained in a previous publication as well as in the S1 File [12]. The derived parameters were based on a Bayesian model fitting exercise using well-informed priors from literature and sexual health survey data [12].

### GetCheckedOnline uptake scenarios

The base case scenario assumed that 4.7% of GBMSM (i.e. susceptible and undiagnosed individuals) used GetCheckedOnline and the remainder used provider-based services based on the observed GetCheckedOnline uptake rate in a community-based survey conducted in 2016 [15]. In the GBMSM who used GetCheckedOnline, 74.1% of high-risk and 43.9% of low-risk non/infrequent testers were assumed to become regular testers in five years (*BC Centre for Disease Control*, *internal data*, *2017)*. The distribution by sexual behaviour and testing patterns would then remain the same for the remaining years although the transitions between sexual behaviours and testing patterns were allowed. Based on the initial distribution by sexual behaviour and testing pattern shown in Table 1, the proportion of low-risk non/infrequent testers would reduce from 16.5% to 9.3% and the proportion of low-risk regular testers would increase from 43.8% to 51.0% in the 5^th^ year and then remain the same. Similarly, the proportion of high-risk non/infrequent testers would reduce from 1.5% to 0.4% and the proportion of high-risk regular testers would increase from 4.0% to 5.1% in the 5^th^ year. In the GBMSM who used clinics, we retained the distribution by sexual behaviour and testing pattern static over the time horizon while allowing the transitions between sexual behaviours and testing patterns. A schematic representation of this scenario is provided in Fig 1 below the model figure. Additionally, two exploratory scenarios were tested to understand the impact of increased GetCheckedOnline uptake (10% and 15% uptake). These exploratory scenarios were chosen based on approximations of results from the Vancouver community survey, in which approximately half of the 32% (i.e., 16%) of GBMSM aware of GetCheckedOnline intended to use it in the future [15].

### Clinical inputs

Progression estimates of HIV-positive patients through post-diagnostic states were derived from HIV natural history research [16, 17]. Individuals’ risk of infection depended on sexual risk behaviour (low or high, depending on type and frequency of sex [12]), while their likelihood of being diagnosed depended on testing pattern (none or infrequent, regular: at least once a year, or frequent: every 3 months). These parameters were derived from program data collected during the first 29 months of GetCheckedOnline implementation (Sept 2014-Jan 2016). Average time of sexual risk activity, rate of sexual encounters, transition rates between sexual risk behaviours, and risk of infection per encounter were derived from estimates published elsewhere [18–21]. Rate of testing and transition rates between testing patterns were based on a cohort study of HIV negative GBMSM in Vancouver, combined with mathematical modeling [12].

The proportions of HIV positive patients under treatment and virally suppressed were derived from provincial HIV treatment monitoring reports [14]. Treatment effectiveness to prevent new infections was assumed to be 100% as per published estimates and given that the proportion of individuals who are virally suppressed was included in the model [20]. Death and emigration were not explicitly modelled; however, a turnover rate was fitted to allow patients to leave and enter the model.

### Cost and resource use inputs

Screening costs were based on a prior cost analysis using GetCheckedOnline and BCCDC provincial STI clinic data. A screening event in a clinic comprised the clerk’s time to book an appointment; the nurse time to provide pre-test counselling, request an HIV test, collect the sample, and capture data on charts; the number and type of HIV tests conducted by the laboratory; the clerk’s time to capture test results into the system; and, finally, the nurse time to communicate results. If requested, negative results were assumed to be given by a nurse by phone; nevertheless, this cost was estimated to represent only 4% of total standard of care costs. GetCheckedOnline costs included the same inputs for standard of care, except for appointment booking, pre-test counseling, and results communication (if negative), tasks which are managed by the client using GetCheckedOnline. Note that the GetCheckedOnline sample collection process is through a private laboratory at a fixed rate. In this model, resource utilization for negative, indeterminate and positive results communication was weighted according to STI clinic practice. Time spent on conducting partner notification service accounted for the proportion of patients that requested this service and the average number of partners these patients had. Data to estimate average resource utilization in each activity was retrieved from clinic registries and conversations with BCCDC nurses. Overall, See S1 File for more information.

Disease-related costs were derived from local published estimates on drug-related and non-drug related costs [22, 23]. Direct costs for HIV positive patients include non-ART-related costs (including hospitalizations, physician billings, laboratory tests, and non-AIDS related drugs) and ART-related costs (including drugs and administration) [22, 23]. Similarly, the mean cost of healthcare management for the susceptible population (i.e. healthy individuals) was derived from a United Kingdom-based estimate and converted to Canadian dollars (CAD) using the purchasing power parity rate [24, 25].

Where necessary, costs were inflated to 2017 CAD using the health care services component of Consumer Price Index [26].

### Utility inputs

Quality-adjusted life years (QALYs) are commonly used in cost-utility analyses to measure health benefits of new interventions [27]. The QALYs are calculated based on the quality of life (or utilities) and the number of remaining life years. A higher utility equates to a preferable health state, with 1 indicating perfect health and 0 death. One QALY is equivalent to one year of perfect health. General population Canada-based utility estimates were not available to assess HIV-related quality of life. For this reason, utilities were derived from a CUA assessing the cost-effectiveness of expanding HIV testing in low-prevalence, high-income settings [24]. Moreover, given that available utility estimates did not reflect general population preferences related to the awareness of infection, selected utility estimates were adapted to avoid penalizing and best characterize the value of early HIV detection to the general population. Utilities, ranges and assumptions are documented in Table 1 and in S1 File. For example, we assumed that individuals with HIV at the disease stage CD4≥500 or CD4: 200–499 without the awareness of infection had the same utilities as individuals diagnosed and not treated.

### Analysis

The analysis was conducted from a healthcare payer perspective, including direct costs in 2017 CAD. Costs and QALYs were discounted at a 1.5% discount rate as recommended by an existing guideline in Canada [28]. A 30-year time horizon was applied, provided that the mean age of infection was 35 years of age and patients living with HIV had almost the same life span as the general population [29]. In addition, in our scenario analyses, we applied 5, 10, and 20-year time horizons.

The model’s primary outcomes included the incremental costs, the incremental QALYs and the incremental cost-effectiveness ratio (ICER) (i.e., the incremental cost per QALY gained). The secondary outcome was the number of averted cases. Point estimates and 95% credible intervals were presented for every outcome. These were constructed through sampling the posterior parameter distribution 1000 times after a burn-in period of 5000 steps. A cost-effectiveness acceptability curve (CEAC) was used to present the probability of GetCheckedOnline being cost effective compared to clinic-based services across a range of willingness-to-pay thresholds including the cost-effectiveness threshold commonly used in Canada, $50,000/QALY.

To explore the sensitivity of results to specific parameter uncertainty and alternative assumptions, in addition to applying different GetCheckedOnline uptake rates (10% and 15%) and time horizons (5, 10, and 20 years), we conducted other scenario analyses by changing only one model input parameter at a time: 1) In the GBMSM who used GetCheckedOnline, we assumed two scenarios by changing the proportion of high-risk non/infrequent testers becoming regular testers in five years from 74.1% in base case to 50% in one scenario and then changing it to 100% in the other scenario; 2) Similarly, in the GBMSM who used GetCheckedOnline, we assumed that 25% or 75% (instead of 43.9% in base case) of low-risk non/infrequent testers, respectively, would become regular testers in five years; 3) Instead of assuming equal utilities in base case, we first assumed that the utilities for undiagnosed positive individuals at the stage of CD4≥500 or CD4: 200–499 were 5% more than those for individuals diagnosed at the same stage but not treated in one scenario, and then 3% in another scenario; 4) Two different discount rates, 0% and 3%, were applied to costs and QALYs, separately.

### Ethics statement

This study is considered Quality Assurance/Quality Improvement (QA/QI) under Article 2.5 of Canada’s Tri-Council Policy Statement 2 (TCPS2; 2018) and is exempt from institutional ethics review, as confirmed by the University of British Columbia Behavioural Research Ethics Board. The TCPS2 is the Canadian framework for research involving human participants and is applied by all institutions receiving funding from the federal government. At the time of account creation, GetCheckedOnline clients were asked to provide written consent online to the information collected through the service being used for evaluation purposes.

## Results

**Fig 2:**
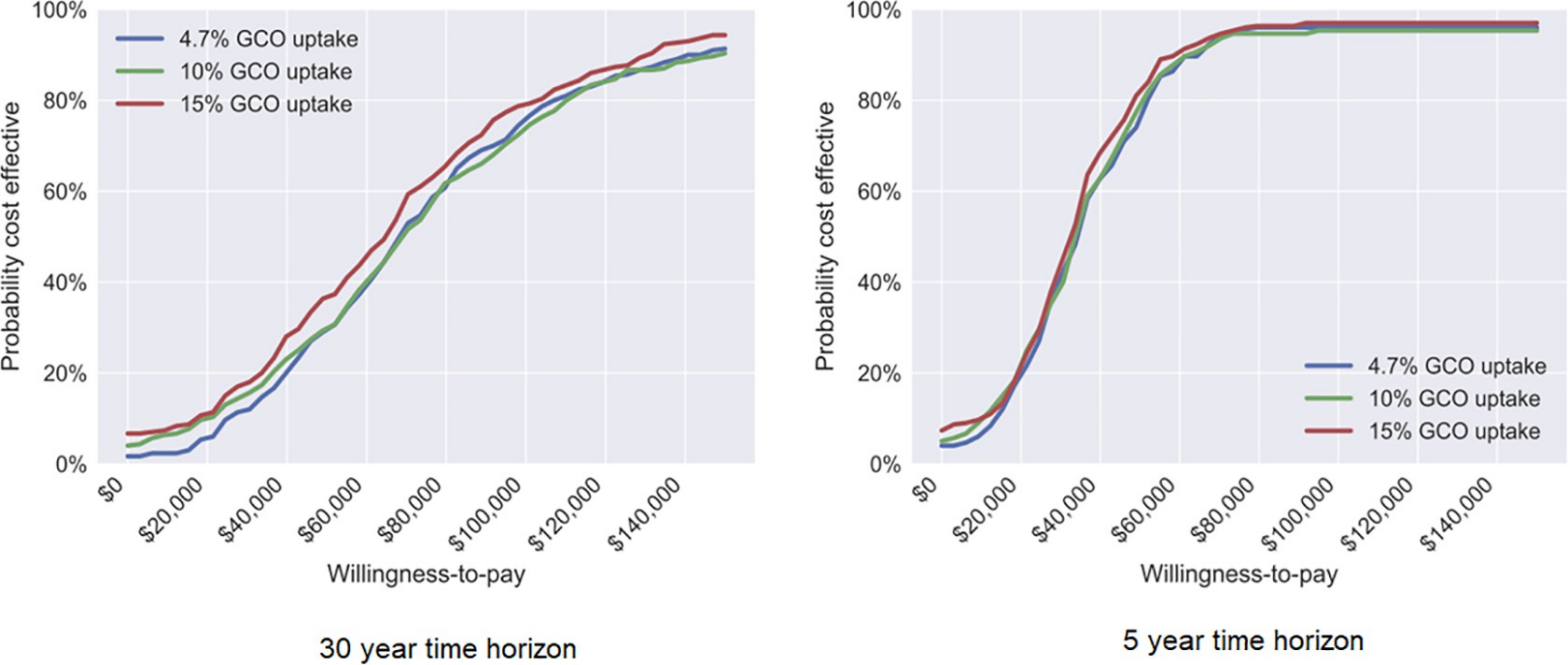
Cost-effectiveness acceptability curve. **Abbreviation:** GCO–GetCheckedOnline.

The cost per HIV screening test was estimated at $29.40 for GetCheckedOnline and $56.92 for clinic-based services (Table 1 and S1 File). Thus, the use of GetCheckedOnline instead of clinic-based services saved about 48% of the cost per HIV screening. The increase in screening frequency and number of patients treated after diagnosis through GetCheckedOnline, however, increased total costs by $329,600 (95% Credible Interval (CrI): -$498,200, $571,000) in a 30-year time horizon in the base case scenario with 4.7% GetCheckedOnline uptake (Table 2). This also translated to 4.53 (95% CrI: 0, 9.20) QALYs gained and 0.94 (95% CrI: 0, 2.31) cases averted. An ICER of $67,000 per QALY (95% CrI: 17,500, 138,600) was achieved. The probability of GetCheckedOnline being cost-effective was 34% at the willingness-to-pay threshold of $50,000 per QALY, reached 50% at the willingness-to-pay threshold of $71,000 per QALY, and increased to 73% at the threshold of $100,000 per QALY in the base case (Table 2 and Fig 2).

**Table 2:** Results of base case analysis and scenario analyses.

Scenario	Time Horizon	Incremental QALYs	Incremental Costs ($)	ICER ($/QALY)	Averted cases	Probability of GCO being cost-effective	
						$50,000/QALY threshold	$100,000/QALY threshold
**Base-case**							
4.7% GCO uptake	30 years	4.53 (0, 9.20)	329,600 (-498,200, 571,000)	67,000 (17,500, 138,600)	0.94 (0, 2.31)	34%	73%
**Scenario Analyses**							
10% GCO uptake	30 years	10.61 (0, 20.82)	721,100 (-1,070,300, 1,266,300)	64,500 (17,900, 134,300)	2.08 (0, 5.04)	37%	80%
15% GCO uptake	30 years	16.62 (0, 31.39)	1,087,200 (-1,520,300, 1,908,100)	63,300 (15,800, 124,600)	3.24 (0, 7.48)	37%	81%
4.7% GCO uptake	20 years	2.71 (0, 5.12)	214,000 (-380,300, 343,500)	73,400 (30,800, 133,700)	0.63 (0, 1.55)	21%	74%
10% GCO uptake	20 years	6.22 (0, 11.38)	462,500 (-806,500, 753,400)	71,300 (30,100, 130,500)	1.39 (0, 3.38)	23%	80%
15% GCO uptake	20 years	9.70 (0, 17.08)	702,100 (-1,151,200, 1,130,700)	70,800 (27,700, 121,600)	2.16 (0, 5.04)	23%	81%
4.7% GCO uptake	10 years	1.10 (0, 1.88)	71,600 (-218,100, 118,400)	61,500 (33,500, 103,000)	0.30 (0, 0.74)	25%	90%
10% GCO uptake	10 years	2.44 (0, 4.16)	154,700 (-456,800, 262,900)	61,200 (33,700, 100,700)	0.66 (0, 1.63)	27%	95%
15% GCO uptake	10 years	3.81 (0, 6.26)	231,600 (-656,900, 405,000)	61,600 (31,700, 94,100)	1.03 (0, 2.42)	27%	93%
4.7% GCO uptake	5 years	0.44 (0, 0.71)	15,000 (-117,400, 34,600)	34,000 (11,300, 63,800)	0.14 (0, 0.34)	80%	98%
10% GCO uptake	5 years	0.98 (0, 1.55)	33,200 (-244,300, 74,500)	33,100 (9,500, 63,100)	0.30 (0, 0.74)	81%	99%
15% GCO uptake	5 years	1.52 (0, 2.35)	50,800 (-351,500, 117,500)	34,400 (9,700, 62,900)	0.47 (0, 1.11)	81%	99%
**50% of high-risk non/infrequent testers becoming regular testers in five years under GCO (it was 74.1% in base case)**							
4.7% GCO uptake	30 years	4.43 (0.0,8.82)	337,000 (-468,989,544,819)	71,450 (23,083,134,157)	0 (0,2)	30%	74%
**100% of high-risk non/infrequent testers becoming regular testers in five years under GCO (it was 74.1% in base case)**							
4.7% GCO uptake	30 years	4.58 (0.0,8.64)	324,251 (-470,924,570,831)	66,132 (17,289,144,406)	1 (0,2)	32%	79%
**25% of low-risk non/infrequent testers becoming regular testers in five years under GCO (it was 43.9% in base case)**							
4.7% GCO uptake	30 years	2.96 (0.0,5.74)	86,097 (-478,071,252,908)	31,079 (-8,194,92,672)	0 (0,1)	68%	93%
**75% of low-risk non/infrequent testers becoming regular testers in five years under GCO (it was 43.9% in base case)**							
4.7% GCO uptake	30 years	7.4 (0.0,15.45)	728,331 (-443,883,1,158,295)	89,627 (34,834,166,098)	1 (0,3)	17%	62%
**5% higher utilities for individuals in disease stage CD4≥500 or CD4: 200–499 without awareness than those diagnosed but not treated (assuming equal utilities in base case)**							
4.7% GCO uptake	30 years	4.08 (0.0,8.27)	351,400 (18,800, 600,400)	80,700 (26,400,177,900)	1 (0,2)	27%	71%
**3% higher utilities for individuals in disease stage CD4≥500 or CD4: 200–499 without awareness than those diagnosed but not treated (assuming equal utilities in base case)**							
4.7% GCO uptake	30 years	4.25 (0.0,8.81)	330,600 (-503,100, 569,900)	72,000 (22,900, 166,900)	1 (0,2)	26%	65%
**Discount rate = 0% (1.5% in base case)**							
4.7% GCO uptake	30 years	4.93 (0.0,9.91)	346,600 (-530,500,610,100)	67,300 (23,200, 147,000)	1 (0,2)	34%	73%
**Discount rate = 3% (1.5% in base case)**							
4.7% GCO uptake	30 years	4.54 (0.0,9.4)	331,000 (-490,200,582,900)	71,900 (21,100, 148,500)	1 (0,2)	31%	73%

95% credible intervals in brackets

**Abbreviations:** GCO–GetCheckedOnline; ICER–Incremental cost-effectiveness ratio; QALY–quality-adjusted-life years

**Fig 3:**
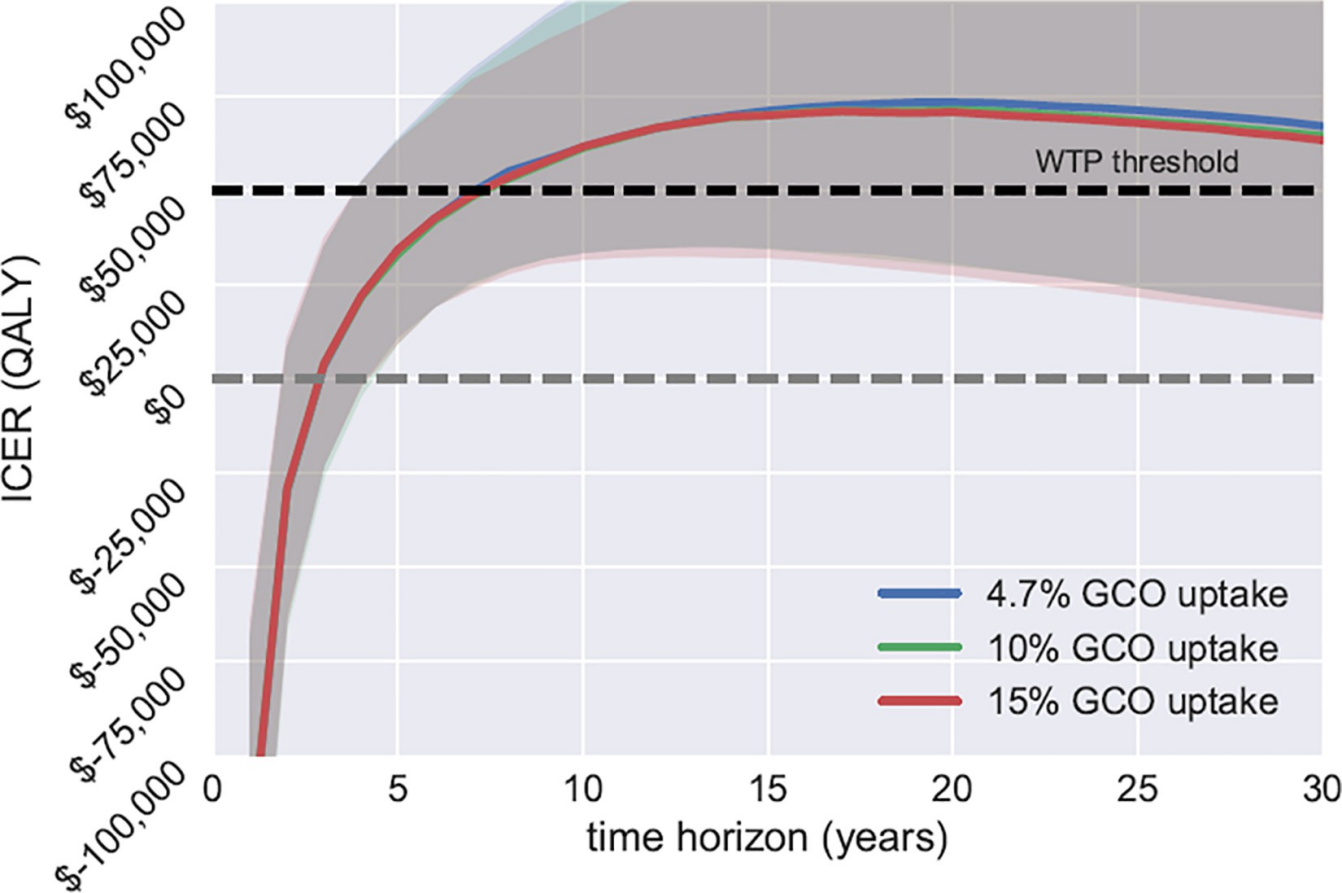
Incremental cost-effectiveness ratio by GetCheckedOnline uptake and time horizon. **Abbreviations:** GCO–GetCheckedOnline; ICER—Incremental cost-effectiveness ratio; QALY—quality-adjusted-life years; WTP—Willingness-to-pay.

Scenario analyses showed that increases in GetCheckedOnline uptake resulted in increased QALYs gained, incremental costs and increased averted cases as expected (Table 2). The ICER and probability of GetCheckedOnline being cost effective did not vary by the GetCheckedOnline uptake rate at the given time horizon (Table 2 and Fig 2). However, the ICERs increased by time horizon until 20 years and then slightly dropped (Fig 3). For example, using 5-year time horizon, the ICER at 4.7% GetCheckedOnline uptake was $34,000 per QALY (95% CrI: 11,300, 63,800), much smaller than the ICER using 10-, 20- or 30-year time horizon. When using 5-year time horizon, the probability of GetCheckedOnline being cost-effective was about 80% at the willingness-to-pay threshold of $50,000 per QALY and about 98% at the threshold of $100,000 per QALY in all three uptake scenarios.

Cost-effectiveness results were not sensitive to variations in the percentage of high-risk non/infrequent testers becoming regular testers in five years within the GetCheckedOnline group, variations in utilities for undiagnosed positive individuals (CD4: 200–499 or ≥ 500), and variations in discount rate (Table 2). However, the probability of being cost-effective increased when there was a lower increase in the overall rate of testing. Assuming only 25% of low-risk non/infrequent testers becoming regular testers in five years, the probability of GetCheckedOnline being cost effective was 68% at the threshold of $50,000 per QALY.

## Discussion

The original implementation of GetCheckedOnline was nearly half as expensive as in-clinic based services for HIV screening on a per-test basis ($56.92 and $29.40 respectively). With no changes in testing behaviours among the population, the program would clearly be cost-saving. However, reduced barriers to testing may potentially lead to individuals with a low risk of acquiring HIV increasing their testing frequency, which may increase costs without further benefit of earlier diagnosis and potential secondary cases averted. Our results suggest that the initial GetCheckedOnline implementation would be a cost-effective strategy to increase screening for GBMSM in Metro Vancouver as compared to standard of care if the willingness-to-pay threshold was over $71,000 per QALY in 30-year time horizon. In a shorter term like 5 years, GetCheckedOnline implementation was cost effective at the commonly used Canadian willingness-to-pay threshold of $50,000 per QALY.

We also showed that cost-effectiveness results were consistent regardless of GetCheckedOnline uptake. Moreover, variations in screening frequencies among the high-risk population were not influential; however, outcomes were highly sensitive to the decrease in testing among the low-risk population (Table 2). This was expected given that the high-risk population that was more likely to acquire and transmit HIV represented only 8% of GBMSM, making screening in this subpopulation more cost-effective, whereas the low-risk population is larger but had a lower prevalence of undiagnosed HIV. Our results suggest that a strategy to favour at least regular screening (i.e. annual) among high-risk population instead of low-risk population could maximize the benefits of GetCheckedOnline expansion, which is consistent with findings from other authors [24, 30–32].

Our evaluation aimed to track as accurately as possible the HIV-related screening and disease management services in Metro Vancouver. However, there were a number of assumptions that were needed to accommodate the complex nature of the disease and the challenging environment of HIV. These included assumptions related to program expectations, disease progression, health states, and quality of life before and after diagnosis.

First, GetCheckedOnline was still in early phases of implementation and expansion, which limited our ability to measure a relative increase in HIV diagnosis rates, and then estimate its cost-effectiveness. Our model estimated the impact of early GetCheckedOnline–in terms of early diagnosis—assuming that increased screening in GetCheckedOnline clients had similar effects to that of in clinic screening [12]. Given that clients from both services are managed by BCCDC, our model assumed that increased and facilitated access to screening would increase the diagnosis rate and that such diagnoses would improve treatment rates, and in turn prevent future cases.

Second, health state severity and disease progression were based on CD4 count only; viral suppression affecting disease progression was not modelled explicitly. This approach has been widely used in HIV-related cost-utility analyses [13, 24, 33]. Furthermore, death rates were not applied directly to the model; instead, a calibrated turnover rate allowed us to model deaths and was applied to every compartment assuming equal likelihood of deaths across health states regardless of disease severity. In this sense, AIDS-related deaths are rarely observed in the province due to early detection and increased ART coverage [30].

Third, certain assumptions needed to be made in the utility inputs to reflect the general population preferences on HIV screening. Although, studies based on HIV positive populations have shown that undiagnosed individuals report higher quality of life than those recently diagnosed, this does not reflect the value of early screening to our society [24]. For this reason, it was assumed that, from the perspective of the general population, the utilities before and after being diagnosed were equal. This assumption, however, did not affect our cost-effectiveness findings as shown by our scenario analyses (Table 2).

The results presented in this study may be generalizable to populations with low prevalence of undiagnosed HIV similar to GBMSM in Metro Vancouver [34]. The prevalence of undiagnosed HIV, prevalence of sexual risk behaviours, and expected frequency of screening play a significant role in determining the cost-effectiveness of programs such as GetCheckedOnline [24]. However, this study was conducted after the first phase of implementation in 2017–2018 based on parameters collected prior to this time, and as such did not account for more recent changes in behaviour. An HIV pre-exposure prophylaxis (PrEP) program launched in BC in 2018, an intervention which can potentially lead to reduced HIV serosorting and potential sorting by use of PrEP among partnerships [35]. The parameterization of sexual behaviour within our study was based on a limited cohort study of 166 individuals, which may potentially bias the resulting frequency of contact and proportion of high to low risk individuals. Furthermore the COVID-19 pandemic has had an extensive impact of both clinical services, and population social and sexual behaviour [36]. Future work should evaluate the impact of GetCheckedOnline within the context of these changes.

In addition, variation of costs may affect cost-effectiveness results. For instance, future loss of exclusivity among patented drugs is likely to improve cost-effectiveness results in the future.

To our knowledge, this is the first economic evaluation on the implementation of an internet-based strategy to expand HIV screening services. Nevertheless, evaluations of other screening services that minimize healthcare professional interactions have reported promising results as well [10, 37]. On the other hand, other studies have analyzed the impact of expanding HIV screening services to increase frequency or broaden target population. Our results coincide with their findings in two dimensions: 1) targeted screening improves cost-effectiveness [24, 38]; and 2) increasing screening frequency to at least annual among targeted groups is a cost-effective measure that, when linked to care, provides the greatest benefit in clinical and economical terms [24, 30–32, 39].

Our model incorporated calibration to a steady state of HIV incidence, which assumes a constant annual incidence. Although the number of HIV diagnoses has remained relatively stable in recent years, changes in testing, treatment, and behaviour including the use of pre-exposure prophylaxis may lead to changes to the rate of HIV incidence and the potential impact of changes in testing services [40].

Our study considered different testing rates stratified by individual risk of HIV acquisition. This segregation helped better understand the relevance of knowing GetCheckedOnline clients’ behaviours. We recognize, however, that in practice it could be difficult to efficiently target clients in this way. Our analysis did not evaluate the impact of the use of internet-based testing in the general population of Metro Vancouver.

GetCheckedOnline was almost half the cost of clinic-based services on a per-test basis. The initial implementation of GetCheckedOnline in Metro Vancouver was found to be a cost-effective strategy to increase screening for HIV among GBMSM. Nevertheless, it must be recognized that our comparator for GetCheckedOnline was based on parameters drawn from a specialized testing clinic, which is perhaps the most resource intensive type of testing, and that a full accounting for costs in other settings accessed by GBMSM for testing in Metro Vancouver (e.g., family physician offices or primary care), may have led to a lower cost-effectiveness estimate. Since the time of our study there have also been other changes to standard of care testing services that may affect our estimates (e.g., online notification of test result systems implemented as a result of COVID). We noted that difference in total costs might be smaller if the battery of STI tests offered by GetCheckedOnline instead of HIV alone is considered, which in turn may affect our cost-effectiveness estimate. For the next phase of analysis, we will expand our model to include testing for additional STIs included in GetCheckedOnline, and to reflect the current phase of implementation. We also recognize that while cost-effectiveness estimates of internet-based testing services for HIV are important it is not the sole measure to determine whether these services should be deployed, given benefits of these services including improving equity in access to testing or reducing the workload and cost for healthcare providers to provide HIV screening.

## Supporting information

S1 File Cost-effectiveness of internet-based HIV screening of HIV in gay, bisexual and other men who have sex with men (GBMSM) in Metro Vancouver, Canada: Full model and methodologies. (PDF)
