# Supplementary material for: Cost-effectiveness of internet-based HIV screening among gay, bisexual and other men who have sex with men (GBMSM) in Metro Vancouver, Canada
Source: PLoS One. 2023 Nov 27;18(11):e0294628. doi: 10.1371/journal.pone.0294628 (PMC10681302; doi:10.1371/journal.pone.0294628)
Supplement: S1 File — (PDF) [file pone.0294628.s001.pdf]

S1 File. Cost-effectiveness of internet-based HIV screening of HIV in gay, bisexual and other men who have sex with men (GBMSM) in Metro Vancouver, Canada: Full model and methodologies

Jose A De Anda[1], Michael A Irvine[2,3], Wei Zhang[1,4,5], Travis Salway[3,6,7], Devon Haag[3], Mark Gilbert[1,3,7]

[1] School of Population and Public Health, University of British Columbia, Vancouver, British Columbia, Canada

[2] Institute of Applied Mathematics, University of British Columbia, Vancouver, British Columbia, Canada

[3] BC Centre for Disease Control, Vancouver, British Columbia, Canada

[4] Faculty of Pharmaceutical Sciences, University of British Columbia, Vancouver, British Columbia, Canada

[5] Centre for Advancing Health Outcomes, Vancouver, British Columbia, Canada

[6] Faculty of Health Sciences, Simon Fraser University, Burnaby, British Columbia, Canada

[7] Centre for Gender and Sexual Health Equity, Vancouver, British Columbia, Canada

Introduction

We implemented a compartmental model based on Irvine et. al., 2018.[1] The model fitting and validation are described in that work. There are a number of notable extensions to the original model including the development of a cost-utility model, to include different stages of disease progression and also the inclusion of a long-term category for individuals living with HIV. For completeness, the full model, including the extended components, are described in full below. Additionally, this supplement describes in detail methodologies used to create costs and utility inputs informing the model.

1. Model description

We implemented a compartmental model of HIV infection in the gay, bisexual, and other men who have sex with men (GBMSM) community in Vancouver, Canada, stratifying individuals according to HIV

infection status, testing pattern, risk group and cohort (a summary of the model is given in the main text Fig. 1). Individuals are assumed to adopt either high or low risk sexual behaviours (indexed by i). Further, each individual's testing pattern is assumed to be one of infrequent/no testing, regular testing or frequent testing (indexed by j). The population is further divided into two cohorts (indexed by l), where an individual is in a standard clinic testing-based cohort ($l = 1$) or an individual is in the GCO testing-based cohort ($l = 2$). Initially, most individuals are susceptible (S_{ijl}) but may become infected at a rate determined by their risk group. Once infected they move to an unaware group that is acutely infected (U_{ijl}^1). An individual can then transfer to the acutely aware group (A_{ijl}^1) at rate τ_j determined by their current testing pattern or they can transition to the latent, unaware group (U_{ijl}^2) at rate ϕ_1. In the latent unaware group (U_{ijl}^2) an individual can either be diagnosed and transition to the latent aware group (A_{ijl}^2) at rate τ_j, or the HIV infection can progress and they transition to the symptomatic unaware stage (U_{ijl}^3) at rate ϕ_2. An individual in the symptomatic unaware stage (U_{ijl}^3) can either be diagnosed at rate τ_j or be hospitalized at rate ϕ_3 and enter the symptomatic aware stage (A_{ijl}^3). A state X_{ijl}^k is therefore the density of individuals in clinical state X, with risk behaviour i, testing frequency j, disease progression k, and cohort l. Given the clinical state, some of these other states will be redundant, such as the disease progression (k) state in the susceptible (S) clinical state or the testing pattern state (j) in the aware (A) clinical state. We, however keep these categories for completeness and compactness of the resulting equations. A proportion of aware individuals will be on treatment and virally suppressed, these are incorporated into the transmission rate as a reduction in the probability of transmission (see below). The corresponding states for a given cohort X_l^k represents a matrix where each entry is the corresponding testing pattern and sexual risk state for that cohort i.e.

$$X_l^k = \left(X_{11l}^k \; X_{12l}^k \; X_{13l}^k \; X_{21l}^k \; X_{22l}^k \; X_{23l}^k\right).$$

There is turnover in the sexually-active population at rate e. This turnover reflects the length of time individuals are sexually active members of the at-risk population. The parameter also captures effects of

emigration and immigration and any other processes that lead to population turnover. An individual can transition away from the sexually active population into a long-term living with HIV and aware (L_A), or long-term HIV negative (L_S) category. As a simplification, it is assumed all individuals with HIV are aware of their status once they move to the long-term category. These categories are necessary to include as individuals may no longer be sexually active or have negligible risk of transmitting or contracting HIV, but still remain in the population and as such their associated costs and benefits need to be accounted for. It is assumed individuals can only transition away from these categories due to death δ. The death rate δ is set such that the combined period of time in the sexually-active and long-term population is equal to the life expectancy of a male living in Canada. We imposed a constant population size constraint by requiring that the rates of entry and exit of individuals to/from the population are equal.

It has previously been shown that episodic risk, where individuals transition between high and low risk, can greatly increase the risk of transmission.[2] We therefore also allow that individuals change their testing patterns or risk group. The rates of these changes form a linear transfer operator T (the details can be found in the Supplementary file of Irvine 2018. As a simplifying step it is assumed that individuals remain in the GCO or clinic-based testing cohort over their lifetime. Different model scenarios are therefore developed where a proportion of the population is moved into the GCO cohort at the start of the campaign.

Costs are accumulated within the model over time. Healthcare-related costs are instantaneously accumulated for an individual dependent on their HIV status and disease progression, whether they are susceptible (C_S) or unaware (C_{U^k}), or aware (C_{A^k}). These costs for individuals is in a long-term category (L) are also considered. The cost for those in L_S is equal to C_S and the cost for those in L_A is assumed to be equivalent to an individual who was diagnosed whilst at acute stage (C_{A^1}). Further costs are accumulated due to cost of the test itself ($C_{test,l}$), which is also dependent on the cohort to which the individual belongs, as well as the rate of testing dependent on the individual's testing group (τ_j). Finally,

there is also an associated one-off cost for positive case management with every new case ($C_{new\ case}$). The costs are discounted with rate γ_c instantaneously through the use of an exponential function.

Similar to costs, QALYs are accumulated over time, with a discount factor γ_Q and dependent on an individual's health state and the corresponding health utility values. Health utility for individuals in the susceptible long-term category is equal to Q_S. Finally, health utility for individuals in the long-term living with HIV category is assumed to be the same as that for individuals who were diagnosed in the acute stage (Q_{A^1}).

Altogether, the model is specified by the following set of ordinary differential equations:

$$\frac{dS_{ijl}}{dt} = \delta(L_A + L_S) - S_{ijl}\beta_i(U,A) + [TS_l]_{ij}, \frac{dU_{ijl}^1}{dt} = S_{ijl}\beta_i(U,A) - (e + \tau_j + \phi_1)U_{ijl}^1 + [TU_l^1]_{ij}, \frac{dU_{ijl}^2}{dt} = \phi_1 U_{ijl}^1 - (e + \tau_j + \phi_2)U_{ijl}^2 + [TU_l^2]_{ij}, \frac{dU_{ijl}^3}{dt} = \phi_2 U_{ijl}^2 - (e + \tau_j + \phi_3)U_{ijl}^3 + [TU_l^3]_{ij}, \frac{dA_{ijl}^1}{dt} = \tau_j U_{ijl}^1 - eA_{ijl}^1 + [TA_l^1]_{ij}, \frac{dA_{ijl}^2}{dt} = \tau_j U_{ijl}^2 - eA_{ijl}^2 + [TA_l^2]_{ij}, \frac{dA_{ijl}^3}{dt} = \tau_j U_{ijl}^3 - eA_{ijl}^3 + [TA_l^3]_{ij}, \frac{dI}{dt} = \sum_{i,j,l} \quad S_{ijl}\beta_i(U,A), \frac{dD}{dt} = \sum_{i,j,l} \quad \tau_j U_{ijl}, \frac{dL_A}{dt} = e\sum_{ijkl} \quad (U_{ijl} + A_{ijl}) - \delta L_A, \frac{dL_S}{dt} = e\sum_{ijkl} \quad S_{ijl} - \delta L_S, \frac{1}{e^{-\gamma_c t}}\frac{dC}{dt} = \sum_{i,j,k,l} \quad C_{U^k}U_{ijl}^k + \sum_{i,j,k,l} \quad C_{A^k}A_{ijl}^k + \sum_{i,j,l} \quad C_S S_{ijl} + C_S L_S + C_{A^1}L_A + \sum_{i,j,k,l} \quad C_{test,l}\tau_j(U_{ijl}^k + S_{ijl}^k) + \sum_{i,j,k,l} \quad C_{new\ case}\tau_j U_{ijl}^k, \frac{1}{e^{-\gamma_q t}}\frac{dQ}{dt} = \sum_{i,j,k,l} \quad Q_{U^k}U_{ijl}^k + \sum_{i,j,k,l} \quad Q_{A^k}A_{ijl}^k + \sum_{i,j,l} \quad Q_S S_{ijl} + Q_S L_S + Q_{A^1}L_A.$$

The transmission model is specified by the vector function $\beta(U,A)$ as follows. We define a mixing parameter ρ that defines how much mixing occurs between the low-risk group (size N_1) and high-risk group (size N_2). Define the mixing vector as $R = (\rho/2, 1 - \rho/2)$ so when $\rho = 0$, there is no mixing between the groups (See Irvine et al 2018). The transmission is a sum over all risk categories and testing groups of both unaware and aware individuals. The contribution towards ongoing transmission is different in the aware and unaware group (represented as p_A and p_U vectors respectively where individuals who are aware they are HIV-positive have different sexual behaviour[3]), as well as if the individual is high or low risk reflected in differing per-encounter risk of transmission . The rate of sexual encounters varies depending on risk group (r_1 for low risk, r_2 for high risk and $r = (r_1, r_2)$). A proportion

of aware individuals are virally-suppressed with probability p_{ART}, effectively removing them from ongoing transmission. Using the Hadamard-Schur product, this can be compactly written as

$$\beta(U, A) = \frac{r}{r_1 \rho N_1 + r_2(1-\rho) N_2} \left(p_U (R \circ r)^T \sum_{j=1}^{3} U_{ij} + p_A (1 - p_{ART})(R \circ r)^T \sum_{j=1}^{3} A_{ij} \right).$$

Each component in the sum is a contribution from each of the risk categories and aware/unaware infection stages to the total force of infection. In order to account for the fact that high-risk individuals are more commonly encountered due to their increased rate of sexual encounter r_i, the transmission rate includes a probability of encountering an individual in a given group weighted by that group's encounter rate.

2. Survey parameter estimation

We used data from a cohort study of HIV negative GBMSM in Vancouver to construct well-informed priors for the model. The study population was recruited from June 18, 2011 to March 2, 2012 at a sexual health centre for GBMSM in Vancouver, Canada. Of the 1141 eligible individuals, 194 consented and 166 completed the baseline survey. Details of the survey methods and findings have been previously published.[4]

Participants completed four surveys and sexual network interviews at baseline and approximately 30, 180 and 360 days after recruitment. The surveys had questions on demographics, HIV testing patterns, personal sexual history, HIV knowledge and attitudes, as well as other questions pertaining to sexual and mental health. The sexual network interviews collected detailed behavioural information for the last sexual encounter with each of up to five most recent sexual partners. The construction procedures of priors from the survey data are detailed below. In general, priors were constructed to take into account the uncertainty in the distribution of parameters and sample size (approximately 1% of population being modelled). The key idea is to construct the priors in such a way that the uncertainty is carried forward into the model predictions so that the posterior reflects the uncertainty within the estimates.

2.1 Transition rates between no, regular and frequent testing

The population is divided into three categories based on their testing behaviour. These testing patterns are defined from the surveys as follows: (i) "no testing" for never or infrequent testing ($\tau_1 = 0.05\ year^{-1}$), (ii) "regular testing" for testing every 6-12 months, ($\tau_2 = 1.66\ year^{-1}$), and (iii) "frequent testing" for testing every three months or more often, ($\tau_3 = 4\ year^{-1}$). From the survey, the proportion in each of these groups is 26%, 44% and 30%, respectively. We also used the survey data to estimate the rates of transitions of individuals between the testing categories by considering the likelihood that individuals reported changes in testing behaviour between the three approximately equidistant time points in the survey (baseline, day 180 and day 360). The raw probability that an individual switched behaviour is then converted into a rate using an exponential waiting time distribution, and the rates are then averaged across time-points.[1] The same method was used to estimate the rates of transitions between low and high risk groups.

2.2 Rate of risky events

We defined the rate of risky events r as the number of sex acts where HIV may be transmitted per unit time. We obtain this parameter value based on participants' responses to survey questions about their five most recent sexual partnerships in the past six months. For each partnership the participants provided the date of the first and last sexual encounter, whether the relationship was ongoing, and the average frequency of sex in the past year. From this information we estimated the number of days of partnership within the last year and the frequency of sex. Since the provided responses to either of these quantities are often vague or approximate, we allow a generous lower and upper bound for each relationship in constructing the prior.

We assume a maximum rate of risky sex acts of once per day on average. Some participants did not report any sex act with their sex partners, so we set the minimum number of sex acts to once per relationship. For each participant and each survey time point we then sum the total number of all risky events over all

their partnerships in the past year, and average this count within a defined risk category to obtain the final estimate.

2.3 Per-encounter risk probability of infection

To calculate the per-encounter risk of infection in the presence of more than one sex act per encounter, we assume that the likelihoods of infection from all risk factors are independent. The probability can then be calculated as

$$
\begin{aligned}
P(infected) &= 1 - P(not\ infected) = 1 - \prod_{x \in sex\ act} P(not\ infected\ by\ x) \\
&= 1 - \prod_{x \in sex\ act} (1 - P(infected\ by\ x)) \\
&= 1 - \prod_{x \in sex\ act} (1 - P(infected | engaged\ in\ x) \times P(engaged\ in\ x))
\end{aligned}
$$

This defines the per-encounter risk of infection for an individual. In order to evaluate this for the population, we split the population into high and low risk groups. This was achieved by defining a force of infection for each participant. The per-partner associated risk was calculated as above ($p_{u,i}^{partner}$) for each participant i in the survey and each partner they reported. This was then multiplied by the rate of sexual encounter for that partner, $r_i^{partner}$. The force of infection for the participant is then defined as $\beta_i = \sum_{partners} r_i^{partner} p_{u,i}^{partner}$. Clustering was performed on the statistic to divide the population into high and low risk groups (see supplementary). The means for each group were used to derive the rate of sexual encounter r_i, the per-encounter risk of transmission of unaware, $p_{u,i}$ and aware, $p_{a,i}$ for the high and low risk group.

3. Model Calibration

A Bayesian inference approach was adopted. This approach allows survey data on sexual behaviour to be combined with literature estimates of per-exposure risk and serological measurements of the annual

incidence. Prior distributions for key model parameters were constructed from questionnaire studies on testing and sexual behaviour.[1]

In order to simplify analysis and increase computational speed, the model was fitted at endemic equilibrium, where the annual incidence of HIV is assumed constant. The likelihood function was constructed from the Poisson distribution, using the annual incidence. Each incidence error was assumed to be independent and identically distributed producing the following likelihood

$$l(x|\lambda,\Theta)=\prod_{i=0}^{n}\frac{\lambda^{x_i}}{x_i!}e^{-\lambda}.$$

Here x_i are the annual incidences and λ is the incidence rate at endemic equilibrium for the model. The posterior for the model $P(\Theta|x)$ is therefore defined as

$$P(\Theta|x)\propto l(x|\lambda,\Theta)P(\Theta).$$

In order to sample from the posterior and produce refined estimates for the parameters incorporating knowledge of the serological data, we adopted a Markov Chain Monte Carlo (MCMC) scheme. Sampling steps occurred using an adaptive Metropolis-Hastings update.[5] Fitting was performed in Python 2.7 using the library PyMC.[6,7] Data processing was carried out using the Pandas library.[8] Data visualization was performed using the libraries Seaborn and Matplotlib.[9] A summary of the model fitted parameters is given in Table 1.

4. Measurement and Valuation of Outcomes

Health-related quality of life (HRQoL) weight or health utility was used to calculate QALYs. It usually range from 0 to 1, where 0 represents death and 1 represents perfect health. Health utility for a given health condition or health state can be indirectly estimated using multidimensional instruments based on population’s preferences.

4.1 Utility Inputs

Canadian population-based utility estimates are not available to assess HIV-related QoL. For this reason, utilities were derived from a CUA assessing the cost-effectiveness of expanding HIV testing in low-prevalence, high-income settings by Long E et al.[10] The authors obtained utility values from other published estimates[11-15] but methodologies to derive point estimates were not described. On the other hand, given that available utility estimates do not reflect general population preferences related to the awareness of infection (i.e., preferring everyone to be diagnosed over undiagnosed), selected utility estimates were adapted to avoid penalizing and best characterize the value of early HIV detection. Utilities, ranges and assumptions were documented in Table 2. For example, we assumed that individuals with HIV at the disease stage CD4≥500 or CD4: 200-499 without the awareness of infection had the same utilities as individuals diagnosed and not treated. To test the sensitivity of results to such assumptions, in our scenario analyses, we assumed that the utilities for undiagnosed positive individuals at the stage of CD4≥500 or CD4: 200-499 were 3% or 5% more than those for individuals diagnosed at the same stage but not treated.

5. Resource Use and Costs

5.1 Resource Utilization Inputs

Screening resource utilization for the susceptible population was based on GCO and STI/HIV BCCDC clinic data. A screening event in standard of care comprises the clerk's time to book an appointment; the nurse time to provide counselling, request an HIV test, collect sample and capture data on charts; the number and type of tests conducted by the laboratory; the clerk's time to capture test results into the system; and, finally, the nurse time to communicate results. Likewise, GCO comprises the same steps except for the resources related to booking, counseling and results communication (whenever negative). Note that GCO sample collection process depends on another provider, hence its cost differs. In this model, resource utilization for negative, undefined and positive results was estimated separately and

overall results communication resource utilization weighted proportionally. In the same way, time spent for conducting partner notification service account for the proportion of patients that request such service and the average number of partners these patients have. Data to estimate average resource utilization in each activity was retrieved from nurses and clerk registries and conversations with BCCDC nurses, depending on data availability. Table 3 and Table 4 show the amount of resources that were considered to estimate screening-related resource utilization.

To obtain accurate estimates of resource utilization for HIV (+) infected patients requires a significant amount of work and resources. Hence, this analysis leveraged from local published estimates on drug-related and non-drug related costs.[16, 17]

It must be said that disease management costs in each compartment related to HIV infected patients were equal for both screening interventions. Thus, the difference in resource use and costs after infection was determined by the time patients spent in each health state (i.e. how early they are diagnosed and prevent a new infection)

5.1.1 Nurses Registry

The nurses registry is a passive paper-based surveillance system that allows the nurses to keep track on the patients' reasons to seek care, diagnosis, treatments, and time spent in each session.

Methodology

A full month (May 2017) was used to analyze data and obtain estimates. Data was transferred to an Excel spreadsheet for analysis.

Data retrieved avoided any kind of personal information including, but not limited to, date of birth, name and social security number. Data obtained included the name of the nurse, room number, date, time of entry, time of completion, reason to seek for care (i.e. STI, HIV, etc.), gender, diagnosis, treatment and other comments.

Time for counselling and sample collection was calculated as the difference between the time of entry and the time completed. Contacts were filtered by gender (only male) and reason to seek care (only HIV-related); additionally, contacts that lasted over 2 hours were considered as outliers and were removed from the analysis. Then, mean time and 95% confidence intervals were calculated.

5.2 Costs Inputs

Unit costs to estimate total screening costs on susceptible and unaware populations are displayed in Table 5. As mentioned above, costs to populate disease management-related health states were derived from local estimates published in the literature. The mean cost of healthcare management for the susceptible population (i.e. healthy individuals) was derived from a UK-based estimate and converted to Canadian dollars using the purchasing power parity rate.[12, 18] Costs for HIV (+) patients include non-HAART-related costs (including hospitalizations, physician billings, laboratory tests, and non-AIDS related drugs) and HAART-related drugs (including drugs and administration).[16, 17] Health states costs in diagnosed population were calculated using models published by Nosyk while health state costs in unaware population assumed a baseline value equal to that of the susceptible population adjusted by Nosyk's model marginal effects depending on CD4 level. Where necessary, costs were inflated to 2017 Canadian dollars using the health care services component of Consumer Price Index.[19]

Table 1. Summary of marginal posterior parameters

Parameter	Description	Posterior median	Posterior 95% credible interval
$p_{u,1}$	Average per-encounter risk among unaware individuals (low risk)	0.003	(0.002,0.004)
$p_{u,2}$	Average per-encounter risk among unaware individuals (high risk)	0.014	(0.012,0.016)
e	Average turnover rate in sexually active-group	0.032	(0.023,0.042)
ρ	Proportion of mixing between high and low risk groups	0.695	(0.575,0.891)
r_1	Average rate of sexual encounter (low-risk group)	51	(44,61)
r_2	Average rate of sexual encounter (high-risk group)	159	(105, 190)
N	Total size of GBMSM population	19813	(17806, 21761)
$p_{a,1}$	Average per-encounter risk among aware individuals (low risk)	0.002	(0.002,0.002)
$p_{a,2}$	Average per-encounter risk among aware individuals (high risk)	0.003	(0.002,0.004)
p_{ART}	Fraction of HIV-diagnosed GBMSM on ART and virally suppressed	0.712	(0.706,0.718)
ϕ_1	Rate of progression from acute to latent stage	4.330	(4.330,4.330)
ϕ_2	Rate of progression from latent to symptomatic stage	0.050	(0.050,0.050)
ϕ_3	Rate of progression from symptomatic stage to hospitalization	0.500	(0.500,0.500)
δ	Average death rate in non-sexually active group	0.020	(0.018,0.027)

Table 2. Utility estimates to derive HRQoL.

Item	Utility	Range	Source
Susceptible	1.00		Fryback, 1993 (20)
Infected (Unaware)			
CD4≥500	0.89	0.85-0.95	Assumption$^{\Psi}$
CD4: 200-499	0.72	0.70-0.80	Assumption†
CD4<200	0.72	0.60-0.75	Long E, 2014 (10)
Diagnosed			
CD4≥500	0.89	0.85-0.95	Long E, 2014 (10)
CD4: 200-499	0.72	0.70-0.80	Long E, 2014 (10)
CD4<200	0.72	0.60-0.75	Long E, 2014 (10)
On HAART			
CD4≥500	0.89	0.85-0.95	Assumption$^{\Psi}$
CD4: 200-499	0.83	0.82-0.87	Long E, 2014 (10)
CD4<200	0.82	0.82-0.87	Long E, 2014 (10)

$^{\Psi}$ Infected patients who remain on CD4≥500 will maintain the same utility regardless of its treatment or disease awareness status

† Unaware patients with CD4 200-499 will have the same utilities as those diagnosed but not under treatment.

Table 3. Resource utilization during screening phase.

Resource	Source	Screening		Negative Results		Positive Result	Retest Needed
		GCO	Clinic	GCO	Clinic		
Clerk: Book appointment time (min)	Clerk records (21)		X				
Nurse: Counselling and sample collection time (min)	Nurses Registry (22)*		X				
Private Laboratory: No. of samples collected	GCO Survey	X					
BCCDC Lab: No. of tests conducted	Lower Mainland Pathology and Laboratory Medicine (23)	X	X				X
Data clerk: Report results in system	Clerk records (21)	X	X	X	X	X	X
Nurse: Time to communicate results (min)	Conversation (24)				X	X	X
Nurse: Time to conduct partner notification	Conversation (24)					X	

*See section 5.1.1 for more details.

Table 4. Resource utilization estimates.

Item	Value	Unit	95% CI	Source
**Results notification**				
% of negative results (STI Clinic)	99%	%		Estimated
% of undefined results (STI Clinic)	1%	%		Assumption
% of negative results (GCO)	97%	%		Estimated
% of undefined results (GCO)	3%	%		Assumption
% of positive results	0.4%	%		STI-IS
Mean time to book appointment	1.89	min per patient	(1.65, 2.13)	BCCDC Data Clerks (21)
Mean time to counsel patient and collect sample	43.33	min per patient	(41.4, 45.3)	BCCDC Nurse registries (22)
Mean time to communicate negative results	2.5	min per result	(2, 3)	BCCDC Nurse (24)
Mean time to communicate undefined results	7	min per result	(4, 10)	BCCDC Nurse (24)
Mean time to communicate positive results	50	min per result	(40, 60)	BCCDC Nurse (24)
Mean time to entry test results GCO	1.1	min per test	(1.05, 1.14)	BCCDC Data Clerks (21)
Mean time to entry test results STI Clinic	0.56	min per test	(0.53, 0.59)	BCCDC Data Clerks(21)
**Partner notification**				
% Clients requesting partner notification	58%	%	(55%, 61%)	BCCDC Nurse (24)
Mean number of partners	20	count	(5, 30)	BCCDC Nurse (24)
Mean time spent to conduct partner notification	15	min per partner	(10, 30)	BCCDC Nurse (24)

Table 5. Unit costs (2017 Canadian dollars).

Item	Cost (2017 CAD)	Unit	Source	Assumptions
Nurse salary	$51.79	cost per hour	NBA Wage Grids. 2014-2019 Provincial Collective Agreement (25)	Assuming most nurses are in the upper end and +22% due to benefits
Clerk salary	$23.23	cost per hour	BCCDC Human Resources	Assuming +22% of benefits
Screening test	$16.23	per test	BCCDC PHL estimates (23)	Assuming the cost of HIV Ag/Ab (Centaur) HIV test and HIV pool NAT STOP HIV
Confirmatory test	$74.26	per test	BCCDC PHL estimates (23)	Assuming the extra-cost for screening patients with evidence of HIV infection
Sample collection (LifeLabs)	$11.75	per sample	Lifelabs Contract (26)	

Table 6. Per event screening costs and health state annual costs (2017 Canadian dollars).

Item	Cost (2017 CAD)	Source	Notes
**Screening Services (cost per event)**			
GCO Screening Services	$29.40	See Table 3	Mean cost per screening event weighted considering potential re-test in undefined results; not including counselling and partner notification services in positive cases
STI Clinic Screening Services	$56.92	See Table 3	
Positive cases management	$267.61	See Table 3	Confirmatory tests, counselling and partner notification services
**Health states (annual cost)**			
General Health Care	$5,409.82	Long EF, 2014 (10)	Converted to Canada by purchasing power parity.
Unaware			
CD4≥500	$6,698.66	Long EF, 2014 (10); Nosyk B, 2015 (17)	
CD4: 200-499	$10,198.62		
CD4<200	$14,294.73		
Off Treatment			
CD4≥500	$7,790.17	Nosyk B, 2015 (17)	
CD4: 200-499	$13,118.04		
CD4<200	$20,260.69		
On Treatment			

CD4≥500	$26,180.25		
CD4: 200-499	$29,544.32	Nosyk B, 2014 (16); Nosyk B, 2015 (17)	
CD4<200	$38,267.15		

References

1. Irvine MA, Konrad BP, Michelow W, Balshaw R, Gilbert M, Coombs D. A novel Bayesian approach to predicting reductions in HIV incidence following increased testing interventions among gay, bisexual and other men who have sex with men in Vancouver, Canada. J R Soc Interface. 2018 Mar 14;15(140).

2. Henry CJ, Koopman JS. Strong influence of behavioral dynamics on the ability of testing and treating HIV to stop transmission. Sci Rep. 2015 Apr 22;5:9467.

3. Gilbert M, Taylor D, Michelow W, Grace D, Balshaw R, Kwag M, et al. Sustained Reduction in Sexual Behavior that May Pose a Risk of HIV Transmission Following Diagnosis During Early HIV Infection Among Gay Men in Vancouver, British Columbia. AIDS Behav. 2018 Jul;22(7):2068–78.

4. Grace D, Chown SA, Jollimore J, Parry R, Kwag M, Steinberg M, et al. HIV-negative gay men's accounts of using context-dependent sero-adaptive strategies. Vol. 16, Culture, Health and Sexuality. Taylor & Francis; 2014. p. 316–30.

5. Hastings WK. Monte Carlo Sampling Methods Using Markov Chains and Their Applications. Biometrika. 1970 Apr;57(1):97.

6. Foundation PS. Python language reference, version 2.7. 1995.

7. Patil A, Huard D, Fonnesbeck C. PyMC: Bayesian Stochastic Modelling in Python. J Stat Softw. 2010 Jul 16;35(4):1–81.

8. McKinney W, others. Data structures for statistical computing in Python. In: Proceedings of the 9th Python in Science Conference. Austin, Texas; 2010. p. 51–6.

9. Hunter JD. Matplotlib: A 2D Graphics Environment. Comput Sci Eng. 2007;9(3):90–5.

10. Long EF, Mandalia R, Mandalia S, Alistar SS, Beck EJ, Brandeau ML. Expanded HIV testing in low-prevalence, high-income countries: A cost-effectiveness analysis for the United Kingdom. PLoS One. 2014;9(4):1–12.

11. Sanders GD, Bayoumi AM, Sundaram V, Bilir SP, Neukermans CP, Rydzak CE, et al. Cost-Effectiveness of Screening for HIV in the Era of Highly Active Antiretroviral Therapy. N Engl J Med. 2005 Feb 10;352(6):570–85.

12. Honiden S, Sundaram V, Nease RF, Holodniy M, Laura C, Zolopa A, et al. The Effect of Diagnosis with HIV Infection on Health-Related Quality of Life The effect of diagnosis with HIV infection on health-related. 2012;15(1):69–82.

13. Tengs TO, Lin TH. A meta-analysis of utility estimates for HIV/AIDS. Med Decis Mak. 2002;22(6):475–81.

14. Schackman BR, Goldie SJ, Freedberg KA, Losina E, Brazier J, Weinstein MC. Comparison of Health State Utilities Using Community and Patient Preference Weights Derived from a Survey of Patients with HIV / AIDS. Med Decis Mak. 2002;22:27–38.

15. Holtgrave DR, Pinkerton SD. Updates of cost of illness and quality of life estimates for use in economic evaluations of HIV prevention programs. J Acquir Immune Defic Syndr Hum Retrovirol [Internet]. 1997;16(1):54–62. Available from: http://www.ncbi.nlm.nih.gov/pubmed/9377126

16. Nosyk B, Montaner JSG, Yip B, Lima VD, Hogg RS, STOP HIVAIDS Study Group. Antiretroviral drug costs and prescription patterns in British Columbia, Canada: 1996-2011. Med Care. 2014 Apr;52(4):362–9.

17. Nosyk B, Lima V, Colley G, Yip B, Hogg RS, Montaner JSG, et al. Costs of health resource utilization among HIV-positive individuals in British Columbia, Canada: results from a population-level study. Pharmacoeconomics. 2015 Mar;33(3):243–53.

18. OECD. Purchasing power parities UK-Canada [Internet]. 2014. Available from: https://data.oecd.org/conversion/purchasing-power-parities-ppp.htm

19. Statistics Canada. CANSIM Table 326-0020 [Internet]. 2017 [cited 2017 May 24]. Available from: http://www5.statcan.gc.ca/cansim/a26?id=3260020

20. Fryback DG, Dasbach EJ, Klein R, Klein BEK, Dorn N, Peterson K, et al. The Beaver Dam Health Outcomes Study: initial catalog of health-state quality factors. Med Decis Mak. 1993 Jun;13(2):89–102.

21. BCCDC STI Clinic. Informal time records registered by clerks: booking and capturing data.

22. BCCDC Nurses. BCCDC Nurses Registry. Vancouver;

23. Lower Mainland Pathology & Laboratory Medicine. Cost of HIV screening protocols. Vancouver;

24. De Anda JA. Conversation with a nurse at BCCDC STI Clinic. Vancouver;

25. BC Nurses Union. NBA WAGE GRIDS – LPNs, RNs & RPNs [Internet]. Vancouver; 2019. p. 1–5. Available from: https://www.bcnu.org/Contracts-Bargaining/Documents/NBA_Wage_Grid.pdf

26. Lifelabs Contract.
